# Age-dependent dissociation between sleep-disordered breathing severity and ventilatory impairment in children with Prader–Willi syndrome

**DOI:** 10.3389/fped.2026.1919314

**Published:** 2026-09-30

**Authors:** Mihail Basa, Jelena Visekruna, Tijana Grba, Sanja Cirkovic, Danijela Radivojevic, Bojana Dobric, Marijana Miskovic, Slavica Ostojic, Aleksandar Sovtic

**Affiliations:** 1 Department of Pulmonology, Mother and Child Health Care Institute of Serbia, Belgrade, Serbia; 2 Laboratory of Medical Genetics, Mother and Child Health Care Institute of Serbia, Belgrade, Serbia; 3 Department of Neurology, Mother and Child Health Care Institute of Serbia, Belgrade, Serbia; 4 Faculty of Medicine, University of Belgrade, Belgrade, Serbia

**Keywords:** central sleep apnea, hypoventilation, non-invasive ventilation, obesity, obstructive sleep apnea, Prader–Willi syndrome, sleep-disordered breathing

## Abstract

**Introduction:**

Sleep-disordered breathing (SDB) is highly prevalent in children with Prader–Willi syndrome (PWS), encompassing central, obstructive, and ventilatory abnormalities. However, its longitudinal evolution remains incompletely understood.

**Methods:**

We conducted a retrospective longitudinal study of children with genetically confirmed PWS who underwent serial respiratory polygraphy with concurrent transcutaneous carbon dioxide (TcCO₂) monitoring. Clinical characteristics, therapeutic interventions, and longitudinal respiratory parameters were analyzed using linear mixed-effects models.

**Results:**

Twenty-nine children were included. SDB demonstrated an age-dependent pattern, with central respiratory abnormalities predominating in early childhood and obstructive events becoming more prominent during preschool and school age. In contrast, nocturnal ventilatory impairment was mainly observed in older children and adolescents. Clinical symptoms were independently associated with ENT intervention, whereas higher body mass index was associated with a greater likelihood of requiring non-invasive ventilation, supporting distinct obstructive and ventilatory phenotypes. Although unadjusted analyses suggested increasing TcCO₂ values with age, adjusted mixed-effects models indicated that this relationship was largely explained by obesity and treatment-related factors.

**Conclusions:**

SDB in children with PWS appears to represent an evolving continuum of central, obstructive, and ventilatory abnormalities. Conventional apnea indices alone may not adequately reflect ventilatory impairment, supporting longitudinal assessment of nocturnal gas exchange in addition to standard respiratory event indices.

## 1 Introduction

Sleep-disordered breathing (SDB) is highly prevalent in children with Prader–Willi syndrome (PWS), encompassing central, obstructive, and ventilatory abnormalities (1). These disturbances have been extensively investigated and are recognized as a major contributor to morbidity in this population, with potential cardiovascular, metabolic, and neurocognitive consequences (2, 3). Over time, significant advances have been made in the management of SDB in PWS, with treatment strategies increasingly tailored to the underlying pathophysiology, including oxygen therapy for central disturbances, adenotonsillectomy for obstructive disease, and non-invasive ventilation (NIV) for hypoventilation (2, 4).

The introduction of growth hormone therapy has further influenced the clinical course of PWS, leading to improvements in body composition, muscle tone, and growth, and potentially modifying respiratory function (5, 6). Nevertheless, distinct risk factors for different forms of SDB remain evident. Central SDB is more commonly observed in younger children and is thought to be related to altered ventilatory control and impaired chemosensitivity, whereas obstructive SDB is associated with adenotonsillar hypertrophy and increasing obesity (7–9). In addition, abnormalities in gas exchange during sleep, particularly hypoxemia and hypercapnia, represent important features of respiratory dysfunction in PWS (10).

Despite these advances, longitudinal data capturing the evolution of SDB in children with PWS remain limited. Most available studies are based on cross-sectional or retrospective designs, providing only a static view of a condition that is likely to change over time (11). Longitudinal studies with repeated measurements in the same patients are particularly challenging due to the rarity of the disorder, variability in clinical presentation, and the need for prolonged follow-up, resulting in heterogeneous datasets and limited sample sizes. In clinical practice, several factors may contribute to delayed referral for sleep evaluation, including the absence of standardized screening pathways, limited access to pediatric sleep laboratories, and the prioritization of nutritional and endocrinological management during early childhood. In addition, most available longitudinal studies have focused predominantly on apnea indices, whereas longitudinal changes in gas exchange abnormalities, particularly nocturnal hypoventilation and hypercapnia, remain less well characterized despite their potential clinical relevance. Nocturnal hypercapnia is increasingly recognized as a clinically relevant marker of ventilatory impairment. In children with PWS, where hypothalamic dysfunction, obesity, and impaired ventilatory control coexist, persistent hypercapnia may contribute to cardiovascular and neurocognitive morbidity beyond that attributable to intermittent hypoxemia alone. Longitudinal characterization of hypercapnic burden is therefore essential to better understand disease progression and identify opportunities for timely intervention.

In this context, a better understanding of how respiratory abnormalities evolve across childhood, particularly with respect to oxygenation, ventilation, and clinical management, is essential. We hypothesized that apneic and ventilatory abnormalities in PWS would follow divergent age-dependent trajectories, with apnea burden predominating in early childhood and hypoventilation emerging later in association with increasing BMI. Therefore, the aim of this study was to evaluate longitudinal changes in oxygenation and ventilation parameters in children with PWS, and to explore their relationship with clinical features, treatment strategies, and SDB phenotypes.

## 2 Materials and methods

### 2.1 Study design and setting

This retrospective study was conducted at a national tertiary pediatric center and included children with genetically confirmed PWS between 2015 and 2026. All eligible patients were included. Inclusion criteria were: (1) genetically confirmed PWS; (2) age 0–18 years at the time of the first sleep study; and (3) availability of at least one technically adequate respiratory assessment, consisting of polygraphy with concurrent transcutaneous capnometry whenever feasible, or transcutaneous capnometry alone when polygraphy could not be successfully completed because of poor tolerance. Patients were excluded only when technically adequate respiratory recordings could not be obtained despite repeated attempts because of severe behavioral dysregulation or profound neurodevelopmental disturbance that precluded sensor placement. Children with acute respiratory infections at the time of scheduled assessment were not studied during the acute illness, and sleep studies were postponed until clinical resolution. The study was approved by the institutional ethics committee (protocol code 8/61; date of approval: 30 April 2026), and informed consent was waived due to the retrospective design. Genetic diagnosis was established with simultaneous assessment of methylation status and genomic dosage at chromosome 15q11q13 region, by the use of methylation-sensitive multiplex ligation-dependent probe amplification (MS-MLPA, commercial kit SALSA® MLPA® Probemix ME028 Prader Willi/Angelman, MRC Holland).

SDB evaluation was performed after the diagnosis of PWS was established, using polygraphy with transcutaneous CO₂ monitoring. Although polysomnography is considered the gold standard, polygraphy represents an acceptable and widely used method for the assessment of SDB in pediatric clinical practice. Polygraphy was performed using the Embletta MPR (Embla, Thornton, CO, USA) or the Portable polysomnography system (PolyPro H2, BMC Medical, Beijing, China). Transcutaneous CO₂ monitoring was conducted using the SenTec Digital Monitoring System (SenTec AG, Therwil, Switzerland). Children with genetically confirmed PWS and at least one sleep study performed at our center were included. All recordings were manually reviewed and scored by experienced clinicians trained in pediatric sleep medicine. Signal quality and physiological plausibility were assessed before analysis, and studies with technically inadequate recordings were excluded. Also, patients with incomplete medical records or without available sleep study data were excluded. Repeat studies were performed based on clinical indications or treatment response, and some patients had multiple sleep studies, allowing for longitudinal within-subject analysis. Sleep studies were scored according to the American Academy of Sleep Medicine (AASM) pediatric scoring criteria (12). As polygraphy does not include neurophysiological channels, hypopneas were defined as a ≥30% reduction in airflow signal accompanied by an oxygen desaturation of ≥3%, in accordance with AASM pediatric scoring criteria for respiratory polygraphy. Alveolar hypoventilation during sleep is defined as a sustained elevation of transcutaneous carbon dioxide (TcCO₂) > 50 mmHg for more than 2% of total sleep time, as assessed by continuous transcutaneous capnometry (13, 14).

Growth hormone (GH) therapy was initiated in all children unless contraindicated by extreme obesity (BMI > + 3 SD) or severe obstructive SDB requiring otorhinolaryngological (ENT) intervention. GH therapy was temporarily discontinued if new SDB symptoms emerged or existing symptoms worsened, provided that follow-up sleep studies confirmed an increase in obstructive apnea–hypopnea index attributable to adenotonsillar growth. Treatment was resumed after ENT intervention and subsequent sleep study reevaluation. Growth hormone therapy status at the time of each sleep study was included as a time-varying covariate in all linear mixed-effects models.

### 2.2 Management of SDB and nocturnal hypoventilation

#### 2.2.1 Central SDB

Long-term oxygen therapy was initiated in children with central sleep apnea, defined as a central apnea index (cAI) > 5 events/h. The duration of oxygen therapy was individualized and determined based on follow-up sleep studies. In selected cases, treatment decisions were influenced by additional parameters, including oxygen desaturation indices and clinical vulnerability in early infancy.

Acetazolamide was introduced when central apnea persisted or worsened despite oxygen therapy, after excluding neurological and cardiological conditions that could account for hypoventilation or central events.

#### 2.2.2 Obstructive SDB

Tonsilloadenoidectomy was recommended in children with obstructive SDB and adenotonsillar hypertrophy. Indications included severe OSA (AHI > 10 events/h) or moderate OSA (AHI 5–10 events/h) accompanied by significant symptoms (habitual snoring, mouth breathing, nocturnal sweating, daytime sleepiness) or relevant desaturation (nadir SpO₂ < 80% and/or T90 > 2%).

Children with moderate or severe obstructive sleep apnea without hypoventilation were treated with CPAP.

#### 2.2.3 Combined obstructive and hypoventilation patterns

Children with isolated nocturnal hypoventilation or with combined obstructive events and hypoventilation were managed with bilevel NIV.

Final modality selection was individualized based on the predominant pathophysiology and clinical context.

### 2.3 Follow-up and monitoring

Routine SDB evaluation was performed annually in all children. In patients receiving long-term oxygen therapy or NIV, follow-up assessments were conducted every six months during inpatient visits to evaluate residual respiratory events and gas-exchange abnormalities. Device software data were reviewed at each visit, including adherence, unintentional leak, usage patterns, and pressure or ventilation parameters relevant to treatment efficacy.

### 2.4 Statistical analysis

Statistical analysis was performed using JASP (version 0.96.0; JASP Team, 2026). Continuous variables were assessed for distribution and presented as mean ± standard deviation or median with interquartile range (IQR), as appropriate. Paired comparisons were performed using the Wilcoxon signed-rank test, while between-group comparisons were conducted using the Mann–Whitney U test. Associations between categorical variables were assessed using Fisher's exact test. Correlations between continuous variables were evaluated using Spearman's rank correlation coefficient.

Longitudinal trends in oxygenation and ventilation parameters were analyzed using linear mixed-effects models with a random intercept for participant ID to account for within-subject repeated measurements over time. Fixed effects included age (modeled as a continuous variable), body mass index (BMI), growth hormone therapy, oxygen therapy, NIV, and history of ENT intervention. Variance inflation factors were used to assess multicollinearity between covariates.

Multivariable logistic regression analyses were performed to identify independent predictors of therapeutic interventions, including ENT surgery and initiation of NIV. Model performance was assessed using standard diagnostic measures. A two-sided *p* value < 0.05 was considered statistically significant. Analyses were performed on a per-measurement basis for longitudinal models and on a per-patient basis for cross-sectional analyses.

## 3 Results

A total of 29 patients were included (14 females, 15 males), with 70 polygraphy studies and 75 capnometry recordings. The higher number of capnometry recordings reflects the fact that in several instances children were unable to tolerate the complete polygraphy setup because of behavioral difficulties or refusal of sensor placement, while transcutaneous capnometry monitoring could still be successfully performed. These recordings were retained for analyses involving ventilation and gas exchange parameters. The median number of sleep studies per patient was 2 (IQR 1–4), with a mean of 3.1 studies per patient (range 1–10). Eighteen patients (62%) had repeated assessments, enabling longitudinal analysis.

The median age at genetic diagnosis was 10.5 months (IQR 1–58.5), whereas the median age at the first sleep study was 32.5 months (IQR 5.5–106.5). This difference was statistically significant (Wilcoxon signed-rank test, *p* < 0.001), indicating a substantial delay between diagnosis and initial sleep evaluation. A strong positive correlation was observed between age at diagnosis and age at first sleep study (Spearman's *ρ* = 0.815, *p* < 0.001), suggesting that delayed diagnosis was associated with delayed referral for SDB assessment.

Growth hormone therapy was administered in 26 of 29 patients. Among these, treatment was temporarily discontinued in one patient due to adenotonsillar hypertrophy and severe obstructive SDB, and subsequently resumed after surgical intervention. In four patients, therapy was permanently discontinued due to severe obesity with progressive weight gain and associated alveolar hypoventilation requiring NIV. In the remaining three patients, growth hormone therapy was not initiated; all were adolescents with severe obesity at the time of evaluation. The median age at growth hormone initiation was 31 months (IQR 6–84). Age at initiation correlated strongly with age at diagnosis (Spearman's *ρ* = 0.871, *p* < 0.001) and with age at the first sleep study (*ρ* = 0.953, *p* < 0.001). Overall, 67.4% of sleep studies were performed during active growth hormone therapy, whereas 32.6% were performed without growth hormone therapy, including studies conducted before treatment initiation, during temporary treatment interruption, or after permanent discontinuation.

SDB requiring clinical intervention was identified in 16 of 29 patients (55%), while 13 patients (45%) had either normal findings or mild SDB not requiring treatment.

Central SDB was identified in 4 patients. At treatment initiation, the median cAI was 4.8 events/h (IQR 3.1–6.4), with treatment decisions based on the overall clinical context rather than cAI alone. All patients were treated with oxygen therapy, initiated at a median age of 15.5 months (IQR 3.5–43), with a median duration of 21 months (IQR 20–22.5). At treatment initiation, mean SpO₂ ranged from 91% to 98%, SpO₂ nadir from 77% to 90%, ODI from 2.6 to 17.9 events/h, and the percentage of sleep time with SpO₂ < 90% (T90) from 0% to 44%. Oxygen therapy was effective in reducing central apnea burden in 3 patients. In one patient, however, central apnea worsened during follow-up (cAI increased from 9.6 to 42.5 events/h after 6 months of oxygen therapy), prompting initiation of acetazolamide. At the time of acetazolamide initiation, transcutaneous CO₂ monitoring demonstrated normocapnia, and brain magnetic resonance imaging performed as part of the clinical evaluation was unremarkable. After one month of treatment, cAI decreased to 11.5 events/h, and after 12 months to 0.5 events/h, allowing discontinuation of therapy without rebound.

Of the 29 patients, 12 (41.4%) had at least one symptom suggestive of SDB, including snoring, mouth breathing during sleep, or witnessed apneas, while none of the patients with central SDB reported such symptoms. The presence of symptoms was significantly associated with the need for therapeutic intervention (Fisher's exact test, *p* = 0.006), with symptomatic patients having markedly higher odds of requiring treatment (odds ratio 14.0, 95% CI 1.8–124.7), primarily ENT procedures or NIV for obstructive disease and/or alveolar hypoventilation.

Six patients had obstructive SDB without alveolar hypoventilation, attributed to adenotonsillar hypertrophy (median AHI 10.3 events/h, IQR 6.25–14.80), and were treated with adenotonsillectomy (median age 44.5 months, IQR 30.25–67.75). Following ENT intervention, AHI decreased to 5.8 events/h (IQR 4.6–8.6) at early follow-up and to 0.6 events/h (IQR 0.3–3.4) at longer-term follow-up. Five patients showed resolution of oSDB, while one developed persistent disease with associated alveolar hypoventilation requiring subsequent NIV.

In addition, six patients presented with alveolar hypoventilation in the presence of mild-to-moderate obstructive events and were initiated on NIV as first-line therapy. At NIV initiation, the median AHI was 5.75 events/h (IQR 4.05–7.92), and all patients exhibited hypercapnia (median TcCO₂ 62 mmHg, IQR 50.8–64.4). The median age in the NIV group was 172 months (IQR 95.5–192), and these patients were significantly older than those treated with ENT intervention (*p* = 0.04). One patient who underwent both ENT intervention and subsequent NIV was excluded from group comparisons.

Figure 1 illustrates the divergent age-related trajectories of apnea severity and ventilatory impairment.

**Figure 1:**
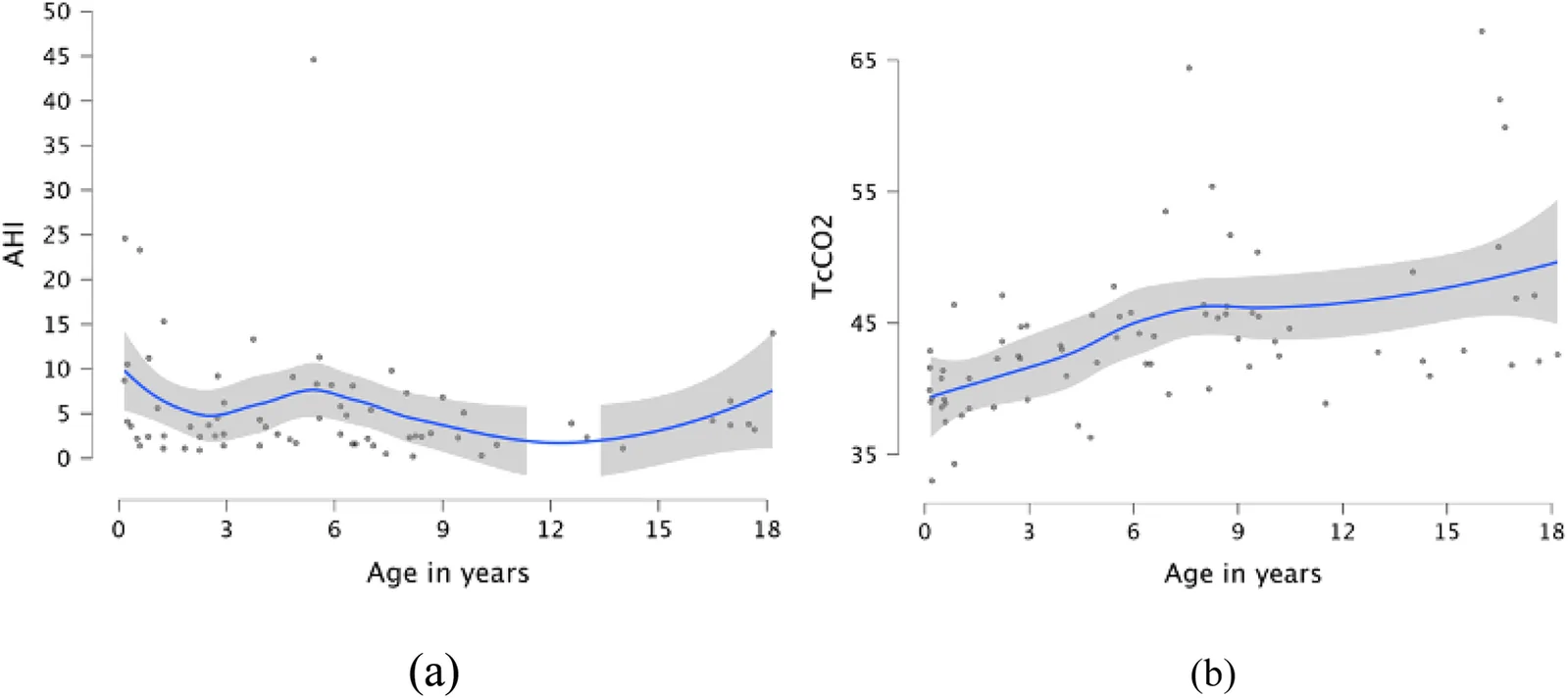
Divergent age-related trajectories of SDB and ventilatory impairment in children with PWS. Scatterplots with LOESS curves (95% CI) show the relationship between age and **(a)** apnea–hypopnea index (AHI) and **(b)** transcutaneous carbon dioxide (TcCO₂). AHI follows a bimodal pattern, while TcCO₂ increases with age, illustrating divergence between SDB severity and ventilatory impairment.

Genetic data were available for 24 patients (13 with deletion of paternal allele of chromosome 15q11q13 region, and 11 with hypermethylation of the differentially methylated sites in PWS/AS critical region suggesting maternal UPD/imprinting defect). No significant association was observed between genetic subtype and the severity of SDB or type of therapeutic intervention (*p* = 0.363).

### 3.1 Longitudinal data

Eleven patients (38%) contributed a single sleep study, while the remaining 18 (62%) had repeated assessments. Given the robustness of linear mixed-effects models to unbalanced longitudinal data under the assumption of missingness at random, all available observations were included in the analyses.

In linear mixed-effects models evaluating nocturnal hypoxemia (% of sleep time with SpO₂ < 90%) and the oxygen desaturation index (ODI), age was not a significant predictor in either model [SpO₂ < 90%: F(1, 22.84) = 0.018, *p* = 0.893; ODI: F(1, 42.08) = 1.049, *p* = 0.312]. BMI showed no significant association with either outcome (all *p* > 0.17), and growth hormone therapy did not influence desaturation burden (all *p* > 0.26). In contrast, children who had undergone ENT intervention exhibited significantly lower hypoxemia and ODI values [SpO₂ < 90%: F(1, 40.30) = 20.699, *p* < 0.001; ODI: F(1, 49.77) = 5.745, *p* = 0.020]. Oxygen therapy during the study was also associated with a significant reduction in both outcomes [SpO₂ < 90%: F(1, 41.29) = 4.907, *p* = 0.032; ODI: F(1, 43.68) = 8.006, *p* = 0.007]. Use of NIV did not show a significant effect in either model (all *p* > 0.30).

In linear mixed-effects models evaluating nocturnal hypercapnia, the association between age and TcCO₂ observed in unadjusted analyses was attenuated after adjustment for BMI, growth hormone therapy, and age at initiation of NIV, suggesting that ventilatory impairment was more strongly influenced by metabolic and treatment-related factors than by chronological age alone. Growth hormone therapy showed a consistent positive association with TcCO₂ levels, although this did not reach statistical significance (mean TcCO₂: *β* = 8.255, *p* = 0.068; maximum TcCO₂: *β* = 10.76, *p* = 0.081). Older age at initiation of NIV was significantly associated with higher TcCO₂ values (mean TcCO₂: *β* = 0.492, *p* = 0.010; maximum TcCO₂: *β* = 0.581, *p* = 0.015).

To further assess the robustness of the longitudinal findings, the linear mixed-effects analyses were repeated in the subgroup of 18 children who underwent at least two sleep studies. Overall, the findings were broadly consistent with those of the primary longitudinal analysis, particularly for ventilatory outcomes. The associations of growth hormone therapy and age at initiation of non-invasive ventilation with mean and maximum TcCO₂ remained statistically significant in the longitudinal subgroup (mean TcCO₂: growth hormone therapy, *p* < 0.001; age at NIV initiation, *p* = 0.004; maximum TcCO₂: growth hormone therapy, *p* < 0.001; age at NIV initiation, *p* = 0.004). The association between age and these outcomes also remained statistically significant (mean TcCO₂: *p* = 0.009; maximum TcCO₂: *p* = 0.011). Growth hormone therapy was also significantly associated with the percentage of sleep time with TcCO₂ > 50 mmHg (*p* = 0.005), whereas the associations of age (*p* = 0.069) and age at initiation of non-invasive ventilation (*p* = 0.050) with this outcome were attenuated. In contrast, the associations of oxygen therapy and ENT intervention with oxygenation-related outcomes (ODI and percentage of sleep time with SpO₂ < 90%) were not reproduced in the subgroup analysis.

Due to the highly skewed distribution of AHI values, the large proportion of near-zero observations, and resulting model instability despite attempted data transformations, mixed-effects analysis was not performed for AHI. Instead, age-dependent AHI trajectories were visualized using locally weighted scatterplot smoothing (LOESS).

### 3.2 Multivariable logistic regression models

Two separate multivariable logistic regression models were constructed (Table 1).

**Table 1:** Multivariable logistic regression analyses identifying predictors of ENT intervention and initiation of NIV in children with PWS.

Variable	ENT intervention OR (95% CI)	*p*-value	NIV OR (95% CI)	*p*-value
**Age**	0.98 (0.96–1.01)	0.131	1.00 (0.98–1.02)	0.871
**Body mass index (BMI)**	1.11 (0.45–2.76)	0.823	19.65 (1.24–311.46)*	**0**.**035**
**Clinical symptoms**	37.56 (1.75–806.26)*	**0**.**020**	0.02 (0.00–2.08)	0.099
**Growth hormone therapy**	5.33 (0.40–71.15)	0.206	0.04 (0.001–2.22)	0.117
**Nagelkerke R^2^**	0.49		0.54	

ENT, otorhinolaryngological intervention; NIV, non-invasive ventilation; OR, odds ratio; CI, confidence interval; BMI, body mass index. Values marked with an asterisk should be interpreted with caution because of the wide confidence intervals, reflecting the limited sample size and the small number of outcome events. Bold values indicate statistical significance (*p* < 0.05).

In the first model, the presence of clinical symptoms was independently associated with the need for ENT intervention (OR 37.56, 95% CI 1.75–806.26, *p* = 0.020), while BMI, age, and growth hormone therapy were not significant predictors. The model yielded a Nagelkerke R^2^ of 0.49.

In the second model, higher BMI was independently associated with the need for NIV (OR 19.65, 95% CI 1.24–311.46, *p* = 0.035), whereas clinical symptoms, age, and growth hormone therapy were not significant predictors. This model yielded a Nagelkerke R^2^ of 0.54.

## 4 Discussion

SDB in PWS demonstrated a dynamic and age-dependent pattern in our cohort, with a continuum from early central abnormalities, through a predominance of obstructive pathology in preschool and early school age, to later ventilatory impairment characterized by alveolar hypoventilation in older children and adolescents. These observations suggest that SDB in PWS should be conceptualized as a time-dependent continuum rather than a set of independent respiratory disorders. This pattern suggests that respiratory control evolves across developmental stages, with distinct contributions of central regulation, upper airway mechanics, and obesity-related ventilatory control.

Despite advances in early genetic diagnosis, a significant delay between diagnosis and initial sleep evaluation was observed, highlighting a potential gap in early respiratory assessment. This delay may limit timely detection of early central respiratory abnormalities and also coincided with delayed initiation of growth hormone therapy in our cohort, reflecting the clinical need for prior respiratory evaluation. This gap underscores the importance of structured respiratory screening at or soon after diagnosis to enable earlier identification of evolving sleep-related breathing disorders (15, 16).

Clinical symptoms were strongly associated with obstructive SDB and the need for therapeutic intervention, particularly in preschool- and school-aged children. In contrast, central SDB and sleep-related hypoventilation were frequently asymptomatic, underscoring the limited value of symptoms as a screening tool for non-obstructive phenotypes (17, 18). Together, these findings support systematic longitudinal respiratory evaluation beyond symptom-driven assessment (19).

The observed age-related distribution of respiratory parameters further supports this phenotypic evolution (4, 20, 21). The bimodal pattern of apnea indices suggests an early peak dominated by central events followed by a second increase driven by obstructive mechanisms, consistent with adenotonsillar hypertrophy (10, 22). In parallel, although adjusted analyses suggested increasing TcCO2 with age, these associations were substantially modified after adjustment for BMI and treatment-related variables, indicating that ventilatory imairment in PWS is influnced by factors beyond chronological age alone (23).

Logistic regression analyses further supported distinct clinical pathways within SDB phenotypes. Clinical symptoms independently predicted ENT intervention, consistent with a predominantly obstructive phenotype. In contrast, NIV was associated with higher BMI and not with clinical symptoms, suggesting that hypoventilation may develop insidiously and remain clinically unrecognized. Oxygenation-related outcomes and ventilatory parameters demonstrated distinct behavior across interventions, highlighting a dissociation between obstructive burden and ventilatory control abnormalities. No significant association was observed between genetic subtype and either therapeutic pathway. Noteworthy, the absence of a statistically significant association in our cohort may reflect the limited sample size and consequent low statistical power, rather than true pathophysiological homogeneity between genetic subtypes. However, previous studies have reported conflicting results regarding the association between genetic subtype and respiratory phenotype in PWS, with some suggesting a higher burden of obstructive SDB in patients with deletion genotypes, while others, consistent with the present findings, have found no clear relationship (24, 25). These discrepancies likely reflect differences in sample size, age distribution, and methodological variability across studies, and the relationship between genotype and respiratory phenotype remains incompletely understood. These findings should be interpreted as exploratory rather than confirmatory. The wide confidence intervals around the odds ratio estimates reflect the limited sample size and relatively small number of outcome events, resulting in considerable uncertainty around the effect estimates. Consequently, these associations should be regarded as hypothesis-generating and require confirmation in larger prospective cohorts.

Central SDB was relatively uncommon and mainly observed in younger patients. Given the very young age of some children, particularly those in early infancy, a relatively permissive approach to supplemental oxygen therapy was adopted, given the uncertainty regarding the short-term evolution of central respiratory events during early infancy in children with PWS. Thus, the decision to initiate oxygen was not based on the CAI alone, but also considered the child's age, overall clinical context, and oxygenation profile. Low-flow oxygen therapy was generally effective in reducing central apnea burden, consistent with its role as a first-line approach (26, 27). However, treatment response was not uniform. In one case, central apnea worsened despite oxygen therapy, prompting introduction of acetazolamide, which led to marked and sustained improvement. Acetazolamide is thought to improve respiratory stability through metabolic acidosis and stimulation of central respiratory drive, and has been used in conditions characterized by altered ventilatory control and impaired chemosensitivity (26, 28). Although evidence in pediatric populations and in PWS specifically remains limited, case reports and small series have suggested potential benefit in selected patients with central SDB (28–30). No standardized recommendations currently exist for its use in this context; therefore, acetazolamide should be considered on an individual basis, with close monitoring of clinical and physiological response (31, 32). These findings support individualized management approaches in selected cases of central respiratory dysfunction.

In contrast, none of the examined interventions, including ENT surgery and oxygen therapy, were associated with improvements in nocturnal hypercapnia. This further emphasizes that ventilatory impairment represents a distinct physiological domain, not directly linked to upper airway obstruction (10). NIV was associated with higher TcCO₂ values, most likely reflecting confounding by indication in more severely affected patients.

Body mass index emerged as the key determinant of nocturnal hypercapnia and the need for NIV. This observation is consistent with the understanding that obesity-related hypoventilation arises not only from increased mechanical load on the chest wall and respiratory muscles, but also from alterations in central respiratory drive, blunted chemosensitivity to carbon dioxide, and leptin resistance (23, 33). In PWS, hypothalamic dysfunction further compounds these abnormalities, as the hypothalamus integrates metabolic and ventilatory control (23). Progressive weight gain may therefore initiate a self-perpetuating cycle in which worsening obesity exacerbates hypoventilation, which in turn reduces physical activity and promotes further weight gain. Recognizing this cycle has direct clinical relevance: interventions that slow or reverse weight gain may help preserve ventilatory function, while early initiation of ventilatory support could prevent the escalation of obesity-related morbidity (8). Overall, ventilatory impairment should be considered a distinct phenotype within SDB rather than a secondary consequence of obstructive disease (34). These findings support routine longitudinal assessment of both obstructive and ventilatory parameters, as symptom-based screening alone is insufficient to identify clinically relevant hypoventilation (35).

Taken together, these findings carry several implications for clinical practice. First, the diagnostic delay between genetic confirmation and initial sleep evaluation suggests that respiratory assessment should be integrated into the standard care pathway at or shortly after diagnosis, rather than deferred until symptoms emerge. Second, the dissociation between symptom presence and ventilatory impairment indicates that clinical history alone is insufficient to exclude significant hypoventilation, supporting periodic objective measurement of gas exchange even in asymptomatic children. Importantly, screening strategies relying exclusively on clinical symptoms or pulse oximetry are insufficient to detect nocturnal hypoventilation, and our findings support the routine integration of transcutaneous capnometry into respiratory monitoring protocols for children with Prader–Willi syndrome. Third, the strong association between BMI and the need for NIV highlights the importance of weight monitoring as a trigger for intensified respiratory surveillance. Based on our findings, respiratory assessment should be considered early after genetic diagnosis and repeated longitudinally, with particular attention to transcutaneous capnometry, as ventilatory impairment may evolve despite relatively mild apnea severity. Although the optimal screening interval remains to be established, closer follow-up appears justified in children with rapid weight gain, documented hypercapnia, or initiation of NIV.

This study has several limitations that should be acknowledged. First, the relatively small sample size reflects the rarity of PWS and limits statistical power for subgroup analyses, particularly when evaluating genotype–phenotype relationships or treatment-specific effects. Second, the single-center and retrospective design, along with non-standardized follow-up intervals, may have introduced bias and limit generalizability. Third, not all patients had repeated measurements, and the number of studies per patient varied considerably, which may have influenced individual trajectories and the overall robustness of the longitudinal analyses. In addition, the absence of electroencephalographic monitoring precluded the detection of cortical microarousals, which may have led to an underestimation of the apnea–hypopnea index, as hypopneas terminated by arousal without significant desaturation could not be scored. This limitation primarily affects the classification of hypopneas and total sleep time duration rather than frank obstructive apneas. Also, it further underscores the importance of routine transcutaneous CO₂ monitoring, as capnometry captures ventilatory impairment independently of respiratory event scoring and may reveal clinically significant hypoventilation even when the apnea–hypopnea index underestimates the true severity of sleep-disordered breathing. In our clinical setting, respiratory polygraphy was selected as the primary diagnostic modality because repeated longitudinal assessments were required and because of the limited availability of pediatric polysomnography. In such contexts, respiratory polygraphy combined with transcutaneous capnometry represents a pragmatic and clinically acceptable alternative, providing sufficiently reliable data on both obstructive events and gas exchange abnormalities to guide clinical decision-making. Noteworthy, detailed data on dietary intake and nutritional interventions were not systematically recorded in this retrospective cohort. While growth hormone therapy and body mass index were included as relevant variables, they do not capture the full complexity of nutritional management in Prader–Willi syndrome. Finally, the observational design precludes causal inference, particularly regarding treatment effects, where confounding by indication cannot be fully excluded. Despite these limitations, the longitudinal design with repeated assessments provides valuable insight into respiratory patterns in children with PWS.

## 5 Conclusion

In children with PWS, SDB appears to represent an evolving continuum of central, obstructive, and ventilatory abnormalities rather than a static condition. Clinical symptoms predominantly reflect obstructive disease and may fail to identify clinically relevant nocturnal hypoventilation. The observed dissociation between apnea burden and ventilatory impairment highlights the importance of longitudinal assessment of gas exchange, including transcutaneous CO₂ monitoring, in addition to conventional apnea indices. Prospective multicenter studies are required to confirm these findings and to define optimal strategies for respiratory monitoring across childhood.

## Data Availability

The raw data supporting the conclusions of this article will be made available by the authors, without undue reservation.
